# Inflammation-related pyroptosis, a novel programmed cell death pathway, and its crosstalk with immune therapy in cancer treatment

**DOI:** 10.7150/thno.62521

**Published:** 2021-08-12

**Authors:** Sheng-Kai Hsu, Chia-Yang Li, I-Ling Lin, Wun-Jyun Syue, Yih-Fung Chen, Kai-Chun Cheng, Yen-Ni Teng, Yi-Hsiung Lin, Chia-Hung Yen, Chien-Chih Chiu

**Affiliations:** 1 Department of Biotechnology, Kaohsiung Medical University, Kaohsiung 807, Taiwan.; 2 Graduate Institute of Medicine, Kaohsiung Medical University, Kaohsiung 807, Taiwan.; 3 Department of Medical Laboratory Science and Biotechnology, Kaohsiung Medical University, Kaohsiung 807, Taiwan.; 4 Graduate Institute of Natural Products, Kaohsiung Medical University, Kaohsiung 807, Taiwan.; 5 Department of Ophthalmology, Kaohsiung Municipal Hsiaokang Hospital, Kaohsiung 812, Taiwan.; 6 Department of Ophthalmology, Kaohsiung Medical University Hospital, Kaohsiung 807, Taiwan.; 7 Department of Biological Sciences and Technology, National University of Tainan, Tainan 700, Taiwan.; 8 Division of Cardiology, Department of Internal Medicine, Kaohsiung Medical University Hospital.; 9 Center for Lipid Biosciences, Kaohsiung Medical University Hospital, Kaohsiung, Taiwan.; 10 Lipid Science and Aging Research Center, Kaohsiung Medical University, Kaohsiung, Taiwan.; 11 Department of Biological Sciences, National Sun Yat-sen University, Kaohsiung 804, Taiwan.; 12 Center for Cancer Research, Kaohsiung Medical University, Kaohsiung, 807, Taiwan.; 13 Department of Medical Research, Kaohsiung Medical University Hospital, Kaohsiung 807, Taiwan.

**Keywords:** Nonapoptotic programmed cell death, pyroptosis, inflammasome, cell death switch, immunotherapy

## Abstract

In recent decades, chemotherapies targeting apoptosis have emerged and demonstrated remarkable achievements. However, emerging evidence has shown that chemoresistance is mediated by impairing or bypassing apoptotic cell death. Several novel types of programmed cell death, such as ferroptosis, necroptosis, and pyroptosis, have recently been reported to play significant roles in the modulation of cancer progression and are considered a promising strategy for cancer treatment. Thus, the switch between apoptosis and pyroptosis is also discussed. Cancer immunotherapy has gained increasing attention due to breakthroughs in immune checkpoint inhibitors; moreover, ferroptosis, necroptosis, and pyroptosis are highly correlated with the modulation of immunity in the tumor microenvironment. Compared with necroptosis and ferroptosis, pyroptosis is the primary mechanism for host defense and is crucial for bridging innate and adaptive immunity. Furthermore, recent evidence has demonstrated that pyroptosis exerts benefits on cancer immunotherapies, including immune checkpoint inhibitors (ICIs) and chimeric antigen receptor T-cell therapy (CAR-T). Hence, in this review, we elucidate the role of pyroptosis in cancer progression and the modulation of immunity. We also summarize the potential small molecules and nanomaterials that target pyroptotic cell death mechanisms and their therapeutic effects on cancer.

## Introduction

Programmed cell death (PCD) plays a significant role in homeostasis. One salient example is apoptosis, which has been well established for several decades. This process participates in, for instance, embryo development, the removal of infected cells, and the clearance of aberrant cells 1, 2. Conversely, the loss of apoptosis might lead to uncontrolled cell proliferation due to a failure to abide by cell cycle checkpoints 3. Hence, in recent decades, apoptosis has been considered an important target in drug development for cancer. For example, curcumin - an antioxidant derived from turmeric - modulates reactive oxygen species (ROS) levels and induces apoptosis by upregulating p53 4. However, increasing evidence has indicated that several types of cancer exhibit chemoresistance due to aberrant apoptosis 5. For instance, evidence from published studies indicates that breast cancer cell lines, such as MCF-7 and MDA-MB-231 cells, are resistant to docetaxel due to elevated expression of miRNA-34a and miR-141 and the suppression of apoptosis 6. Furthermore, Zeinab suggested that ovarian cancer cells exhibit chemoresistance to cisplatin by upregulating miR-221/222 to directly inhibit PTEN (a tumor suppressor) 7. Thus, a strategy that involves bypassing apoptosis and activating nonapoptotic programmed cell death (*e.g.*, pyroptosis) appears promising for cancer treatment.

Pyroptosis is a novel nonapoptotic form of programmed cell death that is strongly associated with the inflammatory response and is also called secondary necrosis 8. It is primarily triggered by inflammasomes and executed by caspases (*e.g.*, caspase-1/-3/-4/-5/-8/-11) and the gasdermin protein family 9-11. Physiologically, pyroptosis serves as an initiator in innate immunity by inducing the release of cytokines, including IL-1β and IL-18, and other molecules, such as high-mobility group protein 1 (HMGB1) and ATP, after cell membrane rupture 12, 13. Intriguingly, accumulating evidence has indicated that pyroptosis plays an important role in the modulation of cancer progression.

For example, an experiment conducted by Qiao and colleagues suggested that 2‐(α-naphthoyl)ethyl‐trimethylammonium iodide (α-NETA), a reversible choline acetyltransferase inhibitor, can suppress the proliferation of multiple epithelial ovarian cancer cell lines by inducing caspase-4-triggered pyroptosis 14. In contrast, Hou *et al.* indicated that programmed death-ligand 1 (PD-L1) translocates to the nucleus of hypoxic cells and upregulates the expression of gasdermin C (GSDMC). This effect eventually contributes to noncanonical caspase-8-mediated pyroptosis in breast cancer but a poor probability of overall survival 11. Additionally, apoptosis shares a similar upstream molecular mechanism with caspase-3-dependent pyroptosis; therefore, the correlation and switch between pyroptosis and apoptosis is worth discussing 15.

In addition to bypassing defective apoptosis, several nonapoptotic cell deaths (*e.g.*, necroptosis and pyroptosis) are highly associated with the immune response; nevertheless, their association with tumorigenesis remains to be elucidated 16.

Furthermore, recent studies have mainly focused on the adaptive immune response and modulation of cancer due to evolutionary discoveries on immune checkpoints, such as programmed cell death protein 1 (PD-1) and cytotoxic T-lymphocyte antigen 4 (CTLA-4), and their application in the clinic; however, the relationship between the innate immune response and cancer treatment has been less investigated 17.

Therefore, in this review, we shed light on the correlation between pyroptosis and its role in antitumor immunity and the modulation of cancer progression.

## Cellular physiology of pyroptosis in host defense

Apoptosis is a form of noninflammatory programmed cell death. In contrast, primary necrosis (accidental cell death) is mainly caused by cellular responses to stresses, such as ischemia, infection, and trauma, and ultimately leads to the release of immunostimulatory components and unmodified damage-associated molecular patterns (DAMPs). In addition, cell swelling and loss of plasma membrane integrity are observed in necrosis 18, 19.

Pyroptosis, a programmed cell death associated with the inflammatory response, is also called secondary necrosis, and secondary necrosis is considered the outcome of complete apoptosis. After apoptosis is initiated, apoptotic cells disintegrate into bodies or vesicles comprising intracellular components and are then phagocytized by scavengers (*e.g.*, macrophages). If apoptotic cells are not removed by phagocytosis, secondary necrosis occurs, despite eat-me signals (engulfment signals expressed on apoptotic cells). Subsequently, this process contributes to a series of events (*e.g.*, cell swelling and a loss of cell membrane integrity) that culminate in plasma membrane rupture and the release of inflammatory stimuli 8, 20, 21. Therefore, Wyllie named this cell autolysis secondary necrosis to distinguish it from primary necrosis 22.

Pyroptotic cell death is primarily observed in professional immune cells (i.e., macrophages and dendritic cells). This process was first described in 1992 when macrophages were observed to execute host cell death after infection with* Shigella flexneri*, a gram-negative bacterium 23.

**Figure 1:**
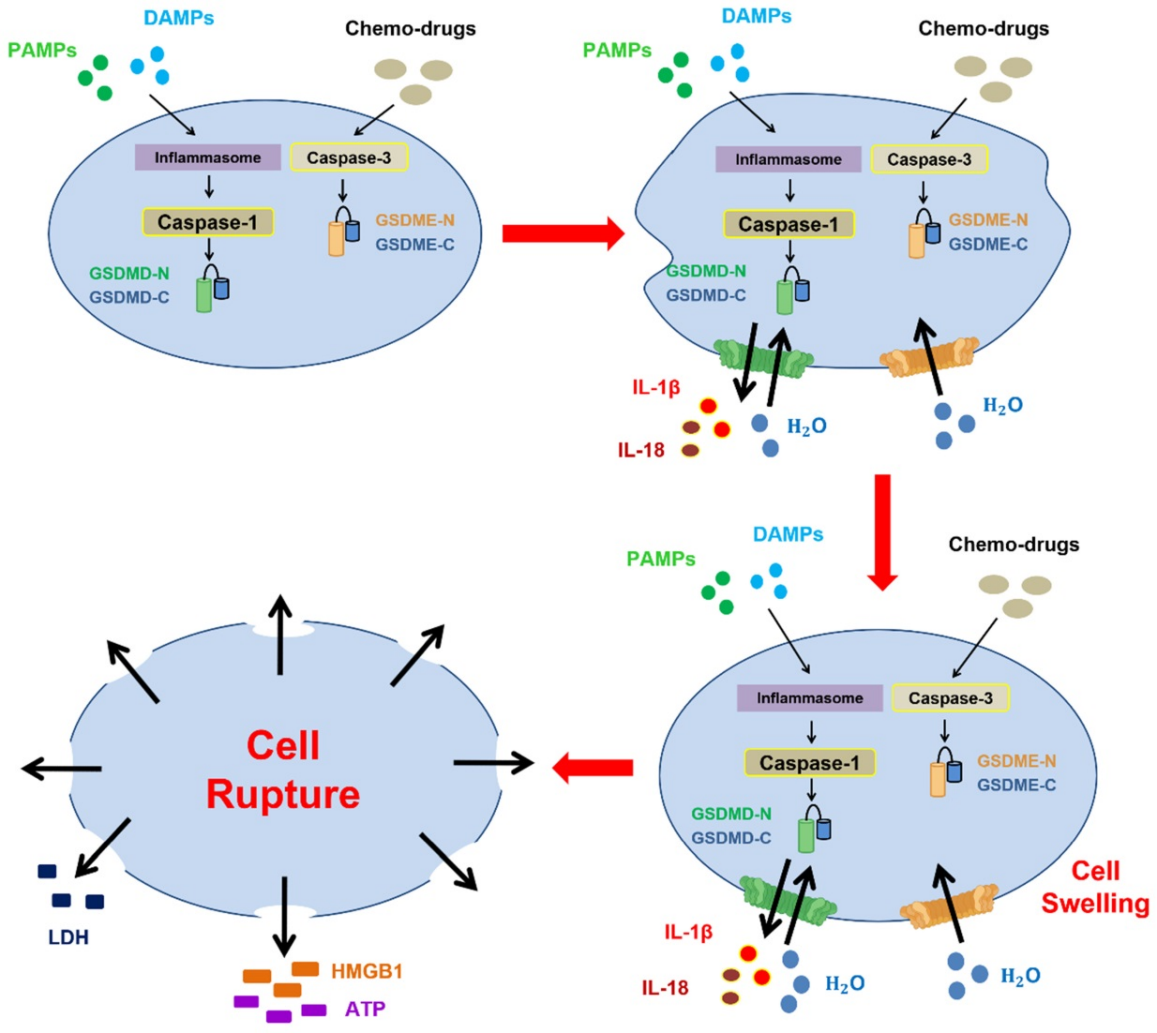
** Morphological changes during pyroptotic cell death.** The inflammasome is activated by PAMPs and DAMPs or chemotherapy, and this activation leads to the activation of caspase-1 or caspase-3, which eventually results in the cleavage of gasdermin D and gasdermin E to form N-terminal fragments, respectively. First, gasdermin-mediated pore formation (nonselective channel) promotes the release of inflammatory substances, including IL-1β and IL-18, due to the large inner diameters of gasdermin pores. Subsequently, the formation of these nonselective channels and the influx of water contribute to cell swelling, membrane blebbing with bubble-like protrusions (known as pyroptotic bodies), continuous cell swelling, and loss of plasma membrane integrity to culminate in rupture of the cell membrane. Finally, HMGB1 and ATP are secreted after cell membrane rupture, followed by pyroptotic cell death.

The characteristics of pyroptosis are described below. (1) Executor proteins (gasdermin family members, N-termini) translocate to the cell membrane and oligomerize to form pores, which is similar to observations of mixed-lineage kinase domain-like pseudokinase (MLKL) in necroptosis. Most gasdermin pores (*e.g.*, gasdermin D) have inner diameters of approximately 10-14 nm 6, 24, and the size of these pores facilitates the secretion of IL-1β and IL-18 (diameters of approximately 4.5 nm), which are involved in several cellular events, such as inflammation, proliferation, and differentiation 13, 24-26. (2) Gasdermin-forming pores are nonselective channels for different ions and promote water influx and cell swelling, in contrast to the formation of selective ion channels and the marked changes in intracellular osmolality observed in necroptosis 27, 28. (3) Continuous water influx is driven by osmotic pressure, further resulting in membrane blebbing with bubble-like protrusions (known as pyroptotic bodies) and the ultimate rupture of the plasma membrane 28. This rupture contributes to the release of DAMPs, such as HMGB1 and ATP, and intracellular contents (*e.g.*, lactate dehydrogenase (LDH)) 6, 12, 29 (Figure 1). Intriguingly, most cell lines with activated inflammasomes exhibit resistance to pyroptotic cell death primarily through the recruitment of endosomal sorting complexes required for transport (ESCRT) on the plasma membrane for repair. The mechanism is initiated by the gasdermin D (GSDMD) pore-mediated influx of calcium Ca^2+^. Hence, the combination of inactive ESCRT-III and transfection with lipopolysaccharide (LPS) leads to increased levels of IL-1β, which indicates that the ESCRT machinery protects cells from the release of proinflammatory cytokines and pyroptosis 30.

The changes in cellular physiology during pyroptosis as a form of pathogen defense are divided into two steps: priming and activation. Before activating the pyroptosis-mediated inflammatory response, the production of related proteins, including inflammasomes and cytokines, is crucial for the initiation of further immune responses. Pathogen-associated molecular patterns (PAMPs), such as components of viruses and bacteria, could promote the generation of type I interferons (IFN-α and IFN-β) for autocrine signaling. Subsequently, type I interferon binds to interferon-alpha/beta receptor 1 and 2 (IFNAR1 and 2), and this binding triggers the JAK/signal transducer and activator of transcription (STAT) signaling pathway and thereby induces the transcription of IFN-stimulated gene (ISG)-encoding proteins to impede pathogens, for instance, to restrict viral replication and degrade viral proteins 31. Moreover, Toll-like receptor (TLR), IL-1 receptor (IL-1R), and tumor necrosis factor receptor (TNFR) activation contribute to the induction of FAS-associated death domain protein (FADD) and caspase-8, which upregulate the NLRP3 inflammasome and pro-IL-1β via the nuclear factor-κB (NF-κB) signaling pathway 32, 33. These components play critical roles in pyroptosis-mediated cell defense.

Notably, the inflammasome - a multimolecular complex - plays an important role in the innate immune system and is involved in inducing pyroptosis 34, 35. In addition to inducing a microbe-mediated immune response, emerging evidence suggests that this complex detects endogenous cellular dangers, such as metabolic stress 36. Activation of the inflammasome is primarily initiated by pattern recognition receptors (PRRs), which are expressed on immune cells (*e.g.*, dendritic cells, macrophages, and neutrophils) and categorized into two forms: membrane-bound PRRs and cytoplasmic PRRs 37, 38. Membrane-bound PRRs are primarily located on the cell surface or the membrane of endocytic compartments and are responsible for recognizing pathogen ligands in extracellular spaces or within endosomes. Cytoplasmic PRRs are located in the cytoplasm and function to detect the presence of intracellular pathogens 39.

PRRs consist of four major subfamilies: Toll-like receptors (TLRs), NOD-like receptors (NLRs), C-type lectin receptors (CLRs), and retinoic acid-inducible gene 1 (RIG-1)-like receptors (RLRs) 38. TLRs and CLRs are mainly membrane-bound PRRs; in contrast, NLRs and RLRs are major components of cytoplasmic PRRs 39.

In particular, NLRs are relatively crucial among the abovementioned PRRs and include the NOD, NLRP, and IPAF subfamilies 37. Several common NLRs, such as NLRP1, NLRP3, NLRC4, AIM2, and pyrin, participate in inflammasome formation. Moreover, inflammasomes are named according to the NLRs in the complex. Specific stimuli activate different PRRs. For example, NLRP1 can sense UVB and toxins secreted by *Bacillus anthracis* 40, 41, NLPR3 recognizes PAMPs and DAMPs 42, AIM2 recognizes cytoplasmic double-stranded DNA 43, NLRC4 detects flagella and type III secretion system 1 (TTSS) expressed by *Salmonella typhimurium* 44, and pyrin senses the bacterial toxin-mediated inactivation of Rho guanosine triphosphatase (Rho GTPase), which eventually reverses the inhibition of immune responses (Table 2) 45.

In general, the inflammasome is mainly composed of PRRs, apoptosis-associated speck-like protein containing a CARD (ASC adaptor, also referred to as PYCARD), and pro-caspase-1. However, some exceptions exist. NLRP1 and NLRC4 directly interact with pro-caspase-1 without mediation by an ASC adaptor, but NLRC4-induced caspase-1 activation is enhanced in the presence of ASC 46-48.

In particular, the NLRP3 inflammasome (that is, part of the NLR is NLRP3) has been extensively investigated, and NLRP3 contains three domains: (1) the pyrin domain (PYD), which interacts with the ASC adaptor; (2) NACHT; and (3) LRR, which functions in ligand sensing and autoregulation 49. ASC bridges NLRP3 and pro-caspase-1 and consists of two domains: (1) PYD, which interacts with NLRP3, and (2) CARD, which interacts with pro-caspase-1 50. Accumulating evidence shows that the unfolded protein response (UPR) can modulate the NLRP3 inflammasome. IRE-1α downstream signaling to c-Jun N-terminal kinase (JNK) plays an important role in activating the NLRP3 inflammasome through the phosphorylation of ASC. Other evidence suggests that C/EBP homologous protein (CHOP) overexpression stimulates the NLRP3 inflammasome 51. In conclusion, pyroptosis plays an important role in host defenses through the inflammasome-induced release of inflammatory components, such as interleukins and DAMPs, during the process of pyroptotic cell death.

## Gasdermin family and molecular mechanism of pyroptosis

### Introduction to gasdermin family members

The gasdermin protein family serves as a central mediator of pyroptosis 52. According to previous studies, this family functions as a tumor suppressor due to its predominant expression in the normal upper gastrointestinal tract but silencing in human gastric cancer cells 53.

The gasdermin family consists of six members: GSDMA, GSDMB, GSDMC, GSDMD, GSDME (also called DFNA5 (deafness, autosomal dominant 5)), and DFNB59 (also known as pejvakin). These members participate in several biological functions, particularly in inducing pore formation on the plasma membrane and pyroptosis 10. In addition, these members are involved in the modulation of epithelial proliferation and differentiation to a certain extent, and in particular, GSDMD has been comprehensively investigated 54.

Most members have similar N-termini (pore-forming domains) and C-termini (autoinhibitory domains) connected by FLTD peptides (linkers) 55. However, their functions in pore formation have not been reported in DFNB59, which has a relatively shorter C-terminus 56. The C-terminus acts as a suppressor by inserting its first loop into the N-terminus. Once the linker is cleaved by specific proteases, for instance, GSDMB is cleaved by granzyme A and GSDMD is cleaved after _272_FLTD_275_ by caspase-1/-4/-5 in humans, the N-terminus is liberated and translocates to the cell membrane 52, 57-59.

The six members of the gasdermin protein family are briefly introduced below.

GSDMA is mainly expressed in epithelial cells of the skin and upper gastrointestinal tract 60. In mice, GSDMA is classified into three gene clusters, namely, *GSDMA*, *GSDMA2*, and *GSDMA3*, due to tandem gene duplication 61. However, the executor protease and specific cleavage sites of GSDMA are poorly understood 62. Emerging evidence has shown that GSDMA is involved in several diseases, such as systemic sclerosis 63.

GSDMB is reportedly associated with autoimmune diseases, such as asthma and Crohn's disease (chronic inflammatory bowel disease) 64, 65. Recent evidence has shown that GSDMB could participate in noncanonical pyroptosis by promoting caspase-4 activity and the cleavage of GSDMD 66. In general, GSDMB is cleaved by granzyme A at Lys^244^/Lys^229^; however, Lys244 is the major cleavage site for granzyme A, and it is sufficient to induce pyroptosis and the clearance of tumors 59.

GSDMC is specifically cleaved by caspase-8 at Asp240 and is related to tumor progression in breast cancer due to the induction of chronic inflammation 11. In addition, Miguchi *et al.* reported that GSDMC functions as an oncogene and promotes colorectal cancer tumorigenesis. The upregulation of GSDMC is accompanied by mutations in APC and transforming growth factor-beta receptor II (TGFBR2), and these features predispose patients to high-frequency microsatellite instability colorectal cancer (MSI-H CRC) 67. Moreover, GSDMC is also considered an unfavorable prognostic indicator for patients with lung adenocarcinoma (LUAD) 68.

GSDMD plays a vital role in inducing pyroptosis, and its mechanism will be discussed in the next section. Shi *et al.* showed that the knockdown of GSDMD by siRNA in mouse immortalized bone marrow-derived macrophages (iBMDMs) leads to the inhibition of pyroptosis and the downregulation of IL-1β even if caspase-1 is intact 58. Nevertheless, GSDMD-mediated pyroptosis is associated with the pathogenesis of several diseases, such as Parkinson's disease (PD) 69.

The mutation of intron 7 in GSDME (DFNA5) is considered the cause of nonsyndromic hearing impairment 70, and recent cancer studies have indicated that its inactivation is to some extent related to gastric cancer 71. Due to its downregulation in several types of cancer, such as breast cancer and hepatocellular carcinoma, GSDME is considered a tumor suppressor 72, 73. GSDME is cleaved by caspase-3 or granzyme B at Asp270 to form pores 74, 75. It also plays a crucial role in switching chemotherapy-induced apoptosis to pyroptosis, depending on the cellular content 76.

Deafness autosomal recessive 59 (DFNB59), also known as pejvakin, is encoded by the *DFNB59* gene. Its mutation is involved in auditory neuropathy, which refers to a hearing disorder in which neural transmission from the auditory nerve to the brain is impaired, although cochlear outer hair cells are intact and functional 77, 78. However, a correlation between pejvakin and cancer has not yet been identified (Table 3).

### Molecular mechanism of pyroptosis

The molecular mechanism of pyroptosis is primarily divided into two pathways: canonical and noncanonical pathways. The canonical pathway depends on caspase-1, whereas the noncanonical pathway is independent of caspase-1 and activated by caspase-4/-5/-11 (caspase-4/-5 in humans caspase-11 in mice), caspase-3, or caspase-8 9, 11.

**Figure 2:**
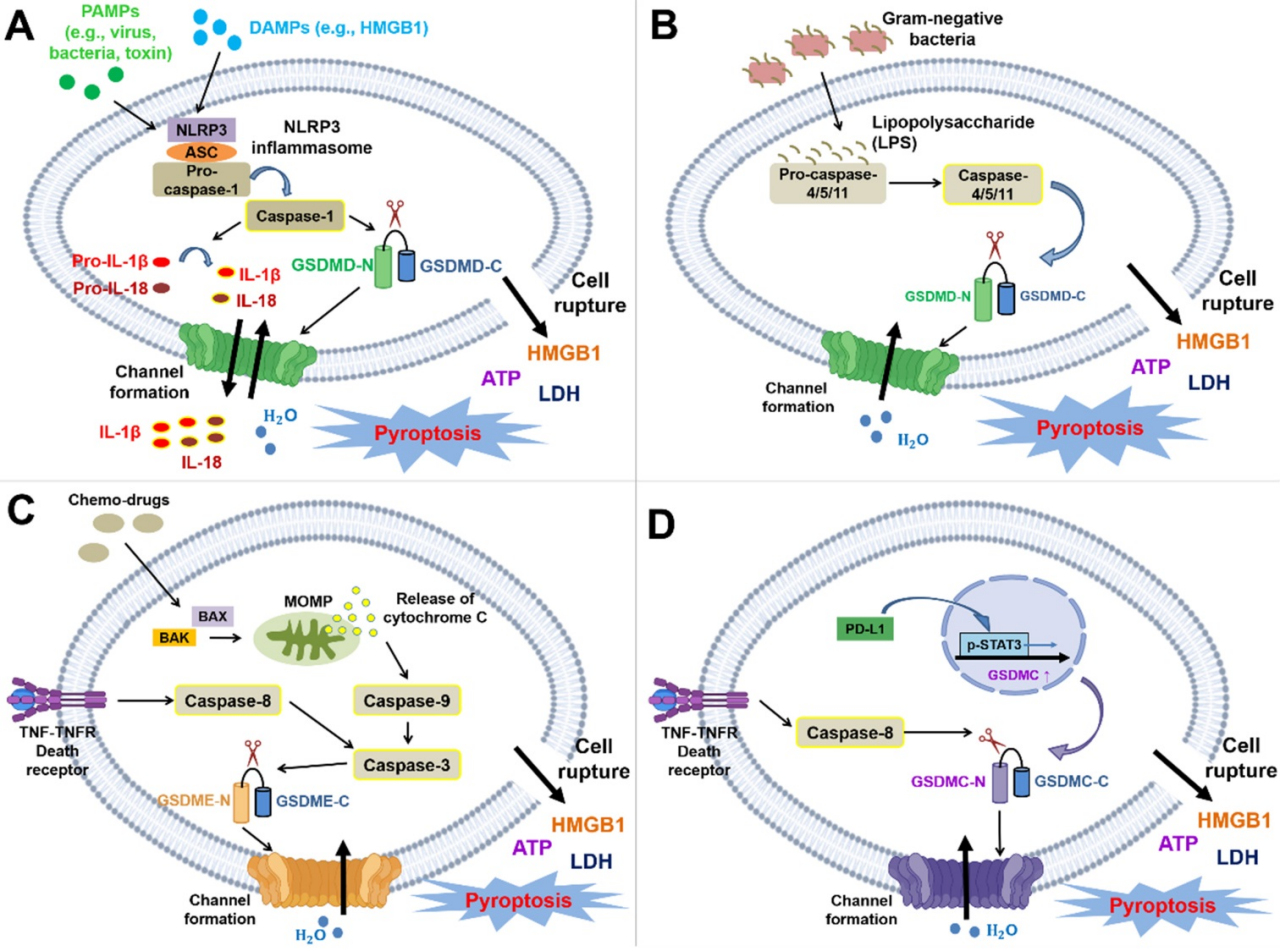
** Molecular mechanisms and cellular physiology of pyroptosis.** The molecular mechanism of pyroptosis is primarily divided into the canonical pathway (dependent on caspase-1) and the noncanonical pathway (dependent on caspase-4/5/11 or mediated by caspase-3 or caspase-8). **(A)** NLRP1, NLRP3, NLRC4, AIM2, and pyrin inflammasomes trigger pyroptosis by inducing the activation of caspase-1. Here, the NLRP3 inflammasome is shown as an example in the figure. The pathway is mainly initiated through the binding of DAMPs or PAMPs to PRRs. PRRs can then recruit the adaptor ASC and pro-caspase-1 to form inflammasomes (the NLRP1 and NLRC4 inflammasomes are exceptions in which ASC is not needed). Procaspase-1 is cleaved by inflammasomes to activate caspase-1, and activated caspase-1 leads to the maturation of IL-1β and IL-18 and the cleavage of GSDMD at _272_FLTD_275_ to generate the N- and C-termini. The N-terminus forms oligomers and translocates to the cell membrane, which finally induces pyroptosis and the secretion of intracellular substances, such as IL-1β, IL-18, HMGB1, ATP, and LDH. Moreover, the released cytokines and DAMPs trigger inflammation and the subsequent immune response. **(B)** The caspase-4/-5/-11-dependent noncanonical pathway is triggered by LPS directly interacting with the CARD of pro-caspase-4/-5/-11 (caspase-4/-5 in humans; caspase-11 in mice). Subsequently, this interaction contributes to the activation of caspase and the cleavage of GSDMD to yield the N- and C-termini. The N-terminus forms oligomers and translocates to the cell membrane to induce pyroptosis. **(C)** The caspase-3-mediated noncanonical pathway is primarily initiated by chemotherapies, mitochondrial dysfunction, or the accumulation of generated ROS. This pathway shares a common upstream signaling pathway with apoptosis; nevertheless, the switch between apoptosis and pyroptosis depends on the cellular content of GSDME. Chemotherapy induces the translocation of BAX/BAK to the mitochondrial outer membrane to form pores, resulting in MOMP and cytochrome C release. Subsequently, this process even sequentially activates caspase-9 and caspase-3. Moreover, caspase-3 is also activated by death receptor signaling-induced caspase-8. Activated caspase-3 can cleave GSDME after Asp270 to generate the C- and N-termini. Similarly, the N-terminus forms oligomers and translocates to the cell membrane to induce pyroptosis. **(D)** GSDMC is upregulated by the PD-L1/p-STAT3 axis and specifically cleaved by death receptor signaling-induced caspase-8. The GSDMC N-terminus forms oligomers and translocates to the cell membrane to induce pyroptosis.

PAMPs and DAMPs mainly initiate the canonical pathway. PAMPs are molecules derived from microbes, such as viral dsDNA, and major components of the fungal cell wall: mannan, β-glucan, and chitin 79. However, LPS in gram-negative bacteria is directly recognized by the CARD of caspase-4/5/11 without the requirement for inflammasomes, which triggers noncanonical pyroptosis 80, 81. DAMPs are molecules from host cells (*e.g.*, tumor cells, dead cells, or cells under cellular stresses), including extracellular proteins (*e.g.*, biglycan and fibrinogen) and intracellular proteins (*e.g.*, histones and heat-shock proteins) 82. PRRs recognize both PAMPs and DAMPs. The interaction of PAMPs or DAMPs with PRRs triggers the signaling pathway and thereby induces the recruitment of ASCs to form inflammasomes, which transform pro-caspase-1 into active caspase-1. Activated caspase-1 (also known as an interleukin-converting enzyme) leads to the maturation of IL-1β and IL-18 and cleaves GSDMD to form C-terminal and N-terminal GSDMD. He *et al.* suggested that GSDMD is essential for the secretion of IL-1β 83. Subsequently, the N-terminus forms oligomers on the inner leaflet of the cell membrane and interacts with phosphatidic acid (PA) and phosphatidylserine (PS) 84; this interaction eventually results in GSDMD-induced pore formation and induces the secretion of IL-1β and IL-18 without the need for cell lysis, cell swelling, bubble formation, and eventually the release of LDH into serum after pyroptosis (Figure 2A) 83, 85, 86. LDH participates in the transformation between pyruvate and lactate and is ubiquitously expressed in cells and tissues. Thus, it has been detected in serum or body fluids after cell damage. A previous study indicated that elevated LDH levels are common in patients with cancer and are associated with a poor prognosis and resistance to treatment 87. LDH is also widely used to detect pyroptosis because it is secreted after cell membrane rupture. However, LDH is insufficient to quantify pyroptosis because LDH is also released during other types of cell death, such as necrosis and apoptosis. Hence, the measurement of LDH levels should be combined with the detection of caspase-1 88.

In the noncanonical pathway, LPS directly interacts with the CARD of caspase-4/-5/-11, which facilitates the active caspase-mediated cleavage of GSDMD. This event results in the generation of C- and N-termini, and the N-terminus forms oligomers and then translocates to the cell membrane inner leaflet 9, 89 (Figure 2B). Chemotherapies primarily induce caspase-3-mediated noncanonical pyroptosis. These chemotherapies trigger apoptotic pathways and pyroptotic pathways, which depend on the expression of GSDME 13. Initially, chemotherapy drugs initiate the activation of BAK and BAX and oligomerization in the mitochondrial outer membrane, which results in mitochondrial outer membrane permeabilization (MOMP) and the release of cytochrome C 90. Subsequently, caspase-9 and its downstream enzyme caspase-3 are activated, and this activation facilitates the caspase-3-mediated cleavage of GSDME after Asp270 to produce the C- and N-termini. In addition, caspase-3 is also activated by death receptor signaling-induced caspase-8 9. The GSDME N-terminus translocates to the plasma membrane and promotes pore formation 15, 28, 74. Based on emerging evidence, GSDME is cleaved by granzyme B at the same site as caspase-3 75 (Figure 2C). Death receptors and their corresponding ligands trigger apoptosis or necroptosis through a process determined by caspase-8; in other words, they share similar upstream signaling pathways 6. Hou *et al.* showed that death receptor signaling is also correlated with pyroptosis. Under hypoxic conditions, TNFα interacts with TNFR, subsequently inducing the activation of caspase-8. Activated caspase-8 specifically cleaves GSDMC at Asp240 to form the GSDMC N-terminus and initiates pyroptosis once GSDMC is upregulated in cells via the PD-L1/pSTAT-3 axis (Figure 2D) 11, 91 In conclusion, the gasdermin family is crucial for executing pyroptosis by inducing pore formation. Pyroptosis is classified into canonical and noncanonical pathways determined by caspase-1 participation.

## Correlation between pyroptosis and modulation of cancer immunity

In addition to noninflammatory apoptosis, several types of nonapoptotic programmed cell death are related to antitumor immunity. For instance, immunotherapy-activated CD8+ T lymphocytes lead to the downregulation of SLC7A11 - a transporter responsible for cysteine intake and subsequent glutathione (GSH) synthesis. Hence, this event facilitates the induction of ferroptosis 92. Concerning necroptosis, necroptotic cells secrete HMGB1 and CXCL1, contributing to the maturation of dendritic cells and CD8+ T lymphocytes 93. Pyroptosis triggers crosstalk between innate and adaptive immunity and modulates the cancer microenvironment to induce an immunostimulatory response.

### Inflammatory cytokines released from pyroptotic cells and their modulatory effects on immunity

One of the characteristics of pyroptosis is the secretion of IL-1β, IL-18, ATP, and HMGB1. Inflammasomes trigger inflammatory responses mainly via two approaches: the secretion of IL-1β and IL-18 by GSDMD-forming pores and the release of HMGB1 and ATP (called DAMPs) after pyroptotic cell rupture 12. A preliminary study showed that IL-1β and IL-18 are important in innate and adaptive immunity; thus, pyroptosis plays a pivotal role in bridging innate and adaptive immune responses 94.

In general, IL-1β is not present in healthy conditions; however, it is primarily secreted by dendritic cells, macrophages, B lymphocytes, and NK cells after activating the immune response. IL-1β can also promote T-cell antigen recognition 95. Additionally, this cytokine reportedly drives the differentiation of naïve CD4+ T lymphocytes to Th17 cells 96. Metastasis is the leading cause of cancer-related death 97. Epithelial-mesenchymal transition (EMT) and mesenchymal-epithelial transition (MET) are necessary processes for the movement of cancer cells from the primary site to distant organs and their subsequent colonization 98. Interestingly, IL-1β reportedly maintains metastatic breast cancer cells along the ZEB1-positive differentiation route, which indicates that IL-1β is capable of inhibiting MET and colonization 99. Hyperactivation refers to the secretion of mature IL-1β from living cells by GSDMD-forming pores 100. Zhivaki *et al.* showed that oxidized phospholipids promote hyperactivation of DCs and the subsequent proliferation of antigen-specific CD8+ T lymphocytes, resulting in CTL-mediated antitumor effects. *In vivo*, basic leucine zipper transcription factor ATF-like 3 batf3-/- mice (lacking hyperactivating DCs) cannot eliminate injected tumors; in contrast, hyperactivating DCs reportedly prevent mice from developing tumors that are sensitive or resistant to anti-PD-1 mAb, which has some benefits on immunotherapy 101.

IL-18 is a key activator of NK and Th1 cells due to the expression of IL18R on the surface of these cells and forms a positive loop with interferon-γ (IFN-γ) 102, 103. IFN-γ exerts antitumor effects by inhibiting the secretion of immunosuppressive cytokines by regulatory T lymphocytes, including transforming growth factor β (TGF-β) and IL-10 104. Furthermore, IFN-γ could trigger the activation and proliferation of CD8+ cytotoxic T lymphocytes and the generation of granzyme B 105. Granzyme B is a serine protease that is expressed at high levels in cytotoxic T lymphocytes and NK cells and eliminates cancer cells by activating apoptotic proteins (*e.g.*, Bid) and degrading antiapoptotic proteins (*e.g.*, Mcl-1)^13^. Additionally, IL-18 leads to the proliferation of NK cells and stimulates the expression of APC-like molecules, such as major histocompatibility complex (MHC) class II and TCR costimulatory molecules, in these cells, which activate Th1 cells and the adaptive immune response 106. Moreover, Hiroaki and his colleagues suggested that IL-18-induced APC-like NK cells derived from patients with lung cancer treated with an anti-EGFR monoclonal antibody exert antitumor effects on the lung cancer cell line PC-9 through antibody-dependent cell-mediated cytotoxicity (ADCC) 106.

As mentioned above, LPS directly triggers caspase-4/-5/-11-mediated noncanonical pyroptosis. Furthermore, activated caspase-11 induces cleavage of the pannexin-1 channel and the release of ATP. In turn, the released ATP activates a P2X7 channel (an ion channel), which leads to cell rupture and potassium (K^+^) efflux. Extracellular K^+^ activates the NLRP3 inflammasome by interacting with NIMA-related kinase 7 (NEK7) and ultimately induces the secretion of IL-1β 107, 108. P2X7 receptors play a pivotal role in modulating immunity within the tumor microenvironment (TME). Marchi *et al.* observed that the tumor sizes in P2X7-null mice are increased due to the immunosuppressive microenvironment, and this increase in size is accompanied by decreased ATP levels and elevated numbers of regulatory T lymphocytes 109. In addition, ATP serves as a major chemoattractant for macrophage migration. Wang and colleagues suggested that the human monocytic leukemia cell line THP-1, when infected with pathogens (i.e., S. aureus or P. aeruginosa) and undergoing pyroptosis instead of only being exposed to bacterial components, can release greater amounts of ATP than apoptotic cells, which facilitates the recruitment of macrophages and even DCs 110.

**Figure 3:**
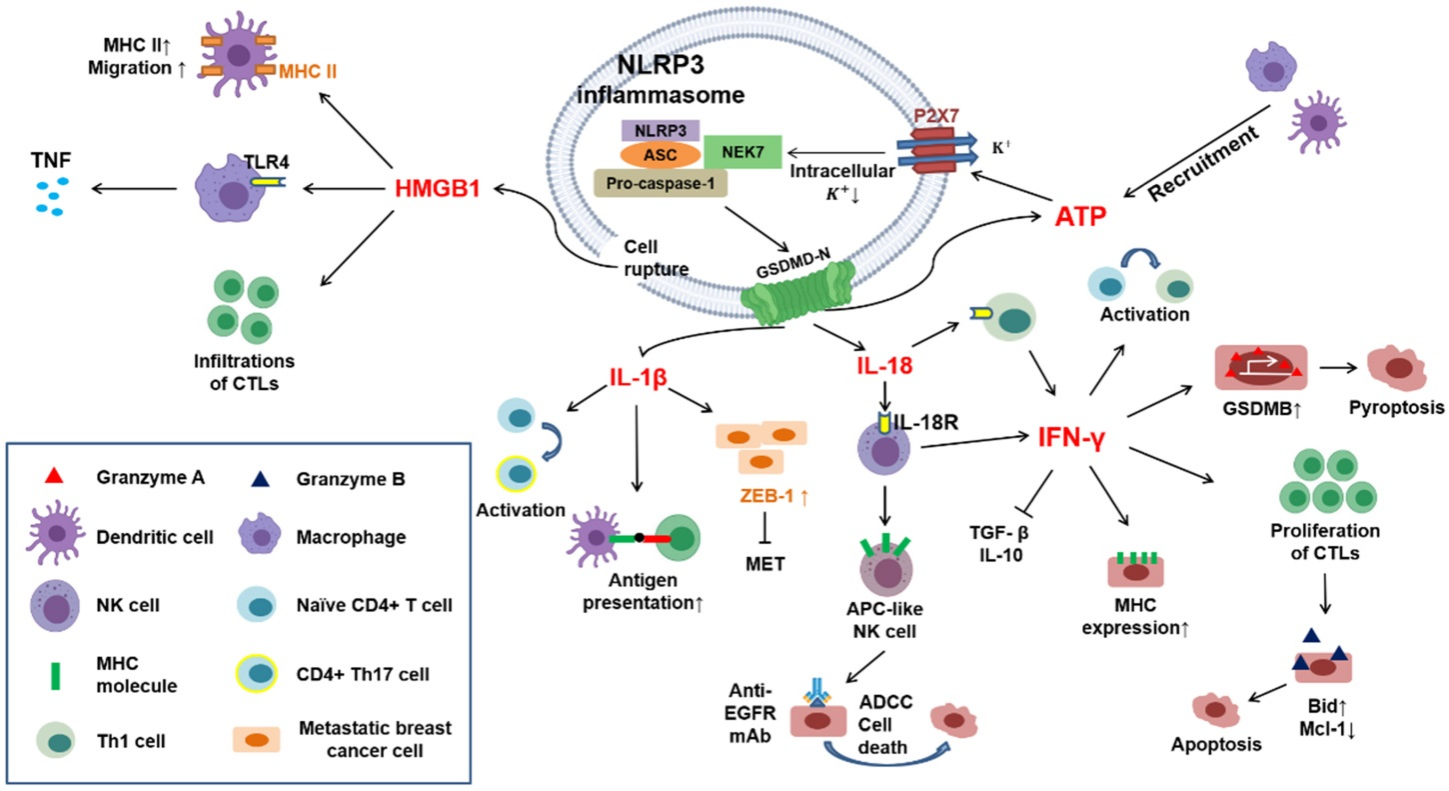
** Crosstalk in immune responses induced by pyroptotic cell death.** Cytokines (*e.g.*, IL-1β and IL-18) and DAMPs (*e.g.*, HMGB1 and ATP) can be released through pyroptotic cell death. IL-1β promotes antigen presentation between dendritic cells and T lymphocytes and drives the differentiation of naïve CD4^+^ T lymphocytes toward a Th17 phenotype. Castano *et al.* suggested that IL-1β can inhibit MET in metastatic breast cancer cells by inducing the expression of ZEB1. IL-18 interacts with IL-18 receptors on immune cells, such as NK cells and Th1 cells, further inducing the generation of IFN-γ. In addition, IL-18 reportedly drives NK cells to DC-like cells through the upregulation of MHC-II and costimulatory molecules. IFN-γ exerts several effects on the activation of the immune response: it can block immunosuppressive cytokines, such as TGF-β and IL-10 secreted by Tregs; it can promote the activation and proliferation of cytotoxic T lymphocytes (CTLs) through the upregulation of granzyme B; it can drive naïve CD4+ T lymphocytes toward a Th1 phenotype; and it can upregulate MHC-II molecules on tumor cells. Furthermore, Zhou *et al.* indicated that IFN-γ upregulates GSDMB in several cancer cell lines, leading to pyroptosis mediated by granzyme A. HMGB1 can upregulate MHC-II molecules on dendritic cells and promote their migration. Moreover, it can induce the secretion of tumor necrosis factor (TNF) from macrophages by interacting with Toll-like receptor 4 (TLR4).

Pyroptosis-induced cell lysis is needed for HMGB1 release, a stimulator of inflammation primarily generated by macrophages and DCs. However, this effect is not observed with IL-1β because GSDMD-mediated pore formation is insufficient for the secretion of HMGB1 12. HMGB1 is a nuclear and cytosolic protein consisting of two folded DNA-binding motifs, box A and box B, and a C-terminus 111, 112. Yang *et al.* demonstrated that HMGB1 leads to the activation of macrophages and the secretion of tumor necrosis factor (TNF) for the innate immune response primarily through interaction with Toll-like receptor 4 (TLR4) 113. Moreover, HMGB1 is also involved in the migration of mature DCs and induces the infiltration of cytotoxic T cells and the upregulation of MHC-II on dendritic cells, promoting antitumor effects 114-116 (Figure 3).

An important point should be further explained. IL-1β and IL-18 secretion is the primary outcome of the canonical pyroptotic pathway. In contrast, in the noncanonical pyroptotic pathway, the release of IL-1β and IL-18 primarily occurs through secondary activation of the canonical pathway because caspases other than caspase-1 are not capable of converting IL-1β and IL-18 into mature forms. Nevertheless, the formation of gasdermin pores mediated by the noncanonical pathway is sufficient to produce ATP and subsequently activate the NLRP3 inflammasome, which ultimately induces IL-1β and IL-18 secretion.

As mentioned before, both pyroptosis and necroptosis induce inflammation; however, some similarities and differences have been identified between necroptosis and pyroptosis-induced immunity. In terms of similarities, both processes trigger the immune response mainly via the release of inflammatory stimuli, for example, the inflammatory cytokines HMGB1 and ATP, which further stimulate bone marrow-derived DC (BMDC) maturation and the production of IFN-γ 93. Moreover, a decrease in the intracellular K^+^ concentration has been observed, and this decrease potentially activates the NLRP3 inflammasome by interacting with NEK7, which eventually contributes to the secretion of IL-1β 108.

Concerning differences, necroptosis is considered a backup cell death process for host defense without caspase-8 activation, regardless of whether caspase-8 is defective or inhibited. In contrast, pyroptosis is a primary cellular immune response to the sensing of DAMPs or PAMPs 27.

### Gasdermin family members and their effect on the modulation of immune cells

NK cells are considered crucial effectors that eliminate tumor cells in innate immunity. These cells recruit mature DCs by releasing CCL5 and CXCL1 and secrete IFNγ to upregulate MHC I molecules on the surface of tumor cells for improved antigen presentation 117. NK cells exert cytotoxicity on target cells via perforin-mediated pore formation 118. It has been widely acknowledged that NK cells kill target cells primarily via the extrinsic apoptotic signaling pathway. Fas family death receptors are expressed on most cells, and NK cells induce apoptosis through a Fas ligand (FasL) interaction with Fas 119. However, apoptosis appears not to be the only type of cell death triggered by NK cell-mediated cytotoxicity. IFNγ secreted by NK cells or CD8+ T lymphocytes reportedly upregulates GSDMB in the esophageal carcinoma cell line OE19, rectum adenocarcinoma cell line SW837, and colorectal adenocarcinoma cell line SK-CO-1. Subsequently, GSDMB is cleaved by granzyme A at Lys^244^/Lys^229^; however, Lys^244^ is the major cleavage site for granzyme A and is sufficient to induce pyroptosis and the clearance of tumors 59. Macrophages are also a vital component of innate immunity due to antigen presentation and the release of cytokines. Macrophage-derived TNFα is reported to induce apoptosis and necroptosis by interacting with death receptors 6. Hou and colleagues suggested that PD-L1 can interact with pSTAT-3 and translocate to the nucleus of hypoxic cells to upregulate GSDMC. Elevated levels of GSDMC result in a switch from TNFα-induced apoptosis to pyroptosis and eventually tumor necrosis 11. Unfortunately, tumor necrosis is related to metastasis and chemoresistance 120; hence, the induction of PD-L1-mediated GSDMC pyroptosis may indicate a poor prognosis for patients with breast cancer 11.

Cytotoxic T lymphocytes (CTLs) play a vital role in immunotherapy by recognizing specific antigens on tumor cells 121. Moreover, CTLs can impose perforin on target cells and deliver granzymes, which leads to pore formation and cytolysis 122; this morphology shares similarities with pyroptotic cell death. Xi *et al.* indicated that the content of GSDMD in CTLs is positively correlated with their responses to the elimination of lung cancer cells. The upregulation of GSDMD in CTLs is consistent with several CD8+ T lymphocyte markers, such as CD8A, GZMB, and IFNG, in activated CTL OT-1. Furthermore, CTL-mediated pyroptosis is mediated by caspase-4; hence, the silencing of caspase-4 by an shRNA attenuates the activation of CTLs and GSDMD-induced pyroptosis in the nonsmall cell lung carcinoma cell line H1299 123. In addition to lung cancer, the downregulation of GSDMD is associated with decreased cytolysis in the ovalbumin-expressing Lewis lung carcinoma cell line 3LL-OVA 123.

**Figure 4:**
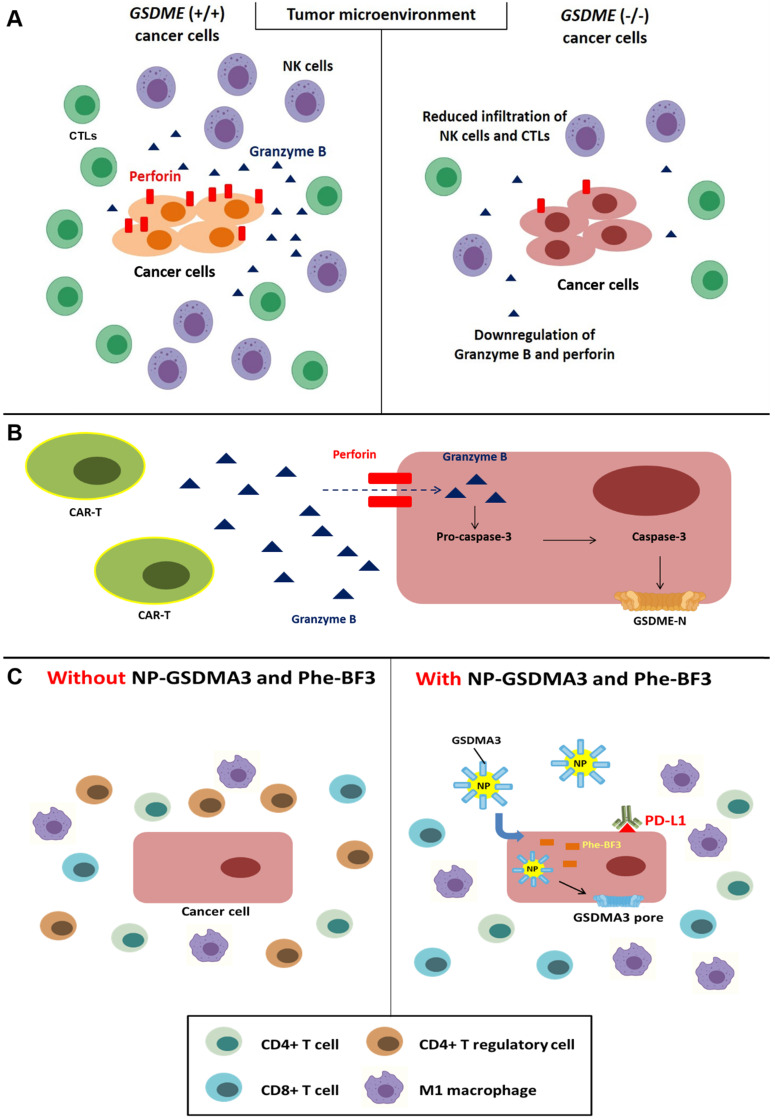
** Correlation between pyroptosis and the tumor immune microenvironment. (A)** Downregulation of perforin and granzyme B and reduced infiltration of NK cells and CTLs were observed in the GSDME^-/-^ cancer cell line microenvironment. **(B)** Caspase-3-mediated pyroptosis in cancer cells is induced through perforin and granzyme B secreted by CAR-T cells. **(C)** The administration of NP-GSDMA3 and Phe-BF3 can induce GSMDA3-mediated pyroptosis in cancer cells and contribute to increased infiltration of immune cells, including CD4^+^ and CD8^+^ lymphocytes and M1 macrophages, and reduced infiltration of CD4^+^ regulatory T lymphocytes.

As mentioned above, GSDME is a key component of caspase-3-mediated pyroptosis. Moreover, this molecule reportedly exerts positive effects on immunity. Reduced infiltration of CD8^+^ lymphocytes and NK cells was observed in the microenvironment of the GSDME^-/-^ murine triple-negative breast cancer cell line EMT6 and colorectal cancer cell line CT26; additionally, this reduction was accompanied by the decreased release of granzyme B and perforin by tumor-infiltrating lymphocytes 75 (Figure 4A). Chimeric antigen receptor T-cell immune therapy (CAR-T) mainly targets refractory CD19^+^ B cell malignancies and has achieved great results 124; however, this therapy might lead to lethal cytokine release syndrome (CRS), including fever and cardiovascular and respiratory insufficiency 125. According to previous research, GSDME is expressed in Burkitt's lymphoma cell line Raji and the acute lymphocytic leukemia (ALL) cell line Nalm-6. In addition, perforin secreted from CAR-T cells leads to granzyme B entry into tumor cells and caspase-3 activation, which triggers the cleavage of GSDME and pyroptosis 126 (Figure 4B). In fact, granzyme B can indirectly induce the cleavage of GSDME via caspase-3 and directly activate GSDME by cleaving the protein after Asp270 75, 127.

### Gasdermin family members and their correlation with immune checkpoint inhibitors (ICIs)

Bioorthogonal chemistry refers to observations of biological behaviors, such as cell death and immunity, under living conditions without cellular toxicity 128. Wang and colleagues established a bioorthogonal system using NP-GSDMA3, which results from the conjugation of gasdermin A3 protein to golden nanoparticles, and the cancer-imaging probe phenylalanine trifluoroborate (Phe-BF3) to investigate the correlation between pyroptosis and immunity. Published evidence indicates that the murine mammary carcinoma cell line 4T1 undergoes pyroptosis after treatment with NP-GSDMA3 and Phe-BF3 (inducing the release of GSDMA3 after NP-GSDMA3 enters cancer cells), and this effect is accompanied by elevated levels of gasdermin N-termini 129, 130. Furthermore, combined treatment with NP-GSDMA3, Phe-BF3, and an anti-PD-1 mAb markedly reduced the tumor size in mice bearing 4T1 tumors, indicating increased sensitivity to anti-PD-L1 cancer immunotherapy. Increased CD4^+^ and CD8^+^ T lymphocyte and M1 macrophage infiltration and reduced numbers of CD4^+^ FOXP3^+^ T regulatory lymphocytes were observed after NP-GSDMA3 and Phe-BF3 administration, and these effects resulted in tumor regression 130 (Figure 4C). In conclusion, the intracellular contents released from pyroptotic cells bridge innate and adaptive immunity by recruiting and activating immune cells. Moreover, this effect positively enhances the efficacy of immunotherapy, including ICIs and CAR-T cells.

### Other inflammasomes and their role in the modulation of immunity

NLPR1 is an inflammasome and is activated by danger-associated signals to trigger inflammation. Gain-of-function mutant *NLPR1* results in a predisposition to skin autoimmune disorders, such as multiple self-healing palmoplantar carcinoma (MSPC) and familial keratosis lichenoides chronica (FKLC) 131. NLRP1 consists of N- and C-termini, and autoproteolysis of the function-to-find domain (FIIND) leads to the release of the NLRP1 C-terminus (NLRP1 CT) for inflammasome activation. Hollingsworth *et al.* indicated that cytosolic dipeptidyl peptidase 9 (DDP9) interacts with full-length NLRP1 and NLRP1 CT to form a ternary complex, which sequesters NLRP1 CT and inhibits inflammation. Conversely, the inhibitory effect is rescued by Val-boroPro (VbP), an inhibitor of DPP8/DPP9 132.

The NLRC4 inflammasome also induces pyroptosis and promotes the secretion of IL-1β and IL-18. According to Lim and colleagues, NLRC4 is upregulated in microglia and astrocytes, resulting in chronic inflammation and glioma (the most prevalent tumor of the CNS). Furthermore, the upregulation of NLRC4 is correlated with a poor prognosis; hence, NLRC4 potentially represents a prognostic indicator 133.

NAD-dependent deacetylase sirtuin-1 (SIRT1) is a histone deacetylase that participates in several biological events, such as cell proliferation and senescence, by epigenetic regulation 134, 135. In addition, SIRT1 plays a significant role in the modulation of immunity. For example, SIRT-1 is activated by AMP-activated protein kinase (AMPK) and then suppresses NF-κB by deacetylating its p65 subunit 136. An experiment conducted by So and colleagues confirmed that SIRT-1 downregulates absent in melanoma 2 (AIM2), which leads to the inhibition of the AIM2 inflammasome and the immune response. Furthermore, SIRT-1 is upregulated in HPV-infected cervical cancer, which facilitates continuous growth 137. However, SIRT-1 knockdown by siRNA contributes to the increased stability of RelB (transcription factor) binding to the AIM2 promoter, promoting AIM2 inflammasome-mediated pyroptosis and inhibiting the growth of the human cervical carcinoma cell line SiHa 138.

Park and colleagues revealed that pyrin inflammasome activation is involved in autoimmune diseases (*e.g.*, familial Mediterranean fever (FMF) and hyperimmunoglobulinemia D syndrome (HIDS)). Missense mutations in *MEFV* (encode pyrin) present in patients with HIDS lead to the spontaneous formation of pyrin inflammasomes 139. Ras homolog (Rho) enhances the activity of protein kinases N1 and N2 (PKN1 and PKN2) that bind and phosphorylate pyrin, leading to the interaction of pyrin and 14-3-3 regulatory proteins to subsequently suppress inflammation. Bacterial toxins inactivate Rho GTPases, resulting in Rho inactivation and activation of the pyrin inflammasome to trigger innate immunity 139.

In addition to the NLRP3 inflammasome, other inflammasomes are involved in modulating immunity and several inflammation-related diseases, such as autoimmune diseases and cancer.

## Relationship between cancer progression and GSDMD-mediated pyroptosis and potential strategies

### Role of GSDMD-induced pyroptosis in cancer progression

GSDMD serves as a double-edged sword in tumorigenesis: on the one hand, it can trigger pyroptosis to facilitate cancer cell death; on the other hand, it can also result in poor prognosis in some cases. For instance, Gao *et al.* indicated that the upregulation of GSDMD is positively correlated with larger tumor sizes and advanced TMN stages in patients with LUAD, indicating a poor prognosis 140.

Preliminary evidence shows that GSDMD is downregulated in gastric cancer cell lines compared with adjacent normal tissue. Conversely, GSDMD reportedly attenuates the transition from the S to G2/M phases of the cell cycle, which results in cell cycle arrest in the gastric cancer cell line BGC823 through the downregulation of cyclinA2 and CDK2. Moreover, GSDMD negatively regulates cell proliferation by inhibiting the extracellular signal-regulated protein kinase 1/2 (ERK1/2), phosphatidylinositol-3-kinase/protein kinase B (PI3K/Akt), and STAT3 signaling pathways 141.

In contrast, GSDMD is upregulated in NSCLC, and this upregulation indicates poor prognosis and advanced TNM stages in patients with lung adenocarcinoma, but no correlation has been found in patients with squamous cell lung cancer. The inhibition of GSDMD attenuates cell proliferation and activates caspase-3 and poly (ADP-ribose) polymerase (PARP) to initiate intrinsic apoptosis in the lung adenocarcinoma cell lines PC9 and H1975 and the squamous lung cancer cell line H1703. In addition, the depletion of GSDMD occurs through the suppression of the EGFR/Akt signaling pathway 140.

Potential biomarkers are crucial for a better prognosis of patients with bladder cancer (BC). CD147, a transmembrane glycoprotein, functions as a regulator of matrix metalloproteinases (MMPs), particularly MMP-2 and MMP-9, through the focal adhesion kinase (FAK)/cortactin signaling pathway, which contributes to cell proliferation, migration, and invasion 142, 143. Peng *et al.* reported a positive correlation between GSDMD and CD147. Elevated GSDMD is consistent with higher expression of CD147 and advanced BC (stage III and IV); in contrast, the inhibition of CD147 in the urothelial cancer cell line T24 leads to reduced GSDMD and better prognosis 144.

### Potential strategies targeting GSDMD-mediated pyroptosis (Table 4)

Schwannomas are tumors derived from different peripheral nerve cell types, such as Schwann cells and axons, and are related to several neurological syndromes (*e.g.*, disability and chronic pain). Tumorigenic transformation is reportedly associated with mutations in the tumor suppressor gene *Nf2* 145. A novel strategy combining adeno-associated virus delivery and the GSDMD N-terminus is suggested to restrict the growth of schwannomas. It was designed with an adeno-associated serotype 1 virus (AAV1)-based vector containing the mouse GSDMD N-terminal gene under the control of the Schwann cell-specific promoter P0. This gene not only shows no neurotoxicity to adjacent tissues after an intratumor injection but also leads to growth suppression in the schwannoma cell lines NF2 and HEI-193 through GSDMD-mediated pyroptosis 146.

Myc reportedly induces tumorigenesis in more than half of human cancers, such as hepatocellular carcinoma and lung cancer 147, 148. Moreover, its activation inhibits the recruitment of CD4+ T lymphocytes, contributing to angiogenesis, a loss of apoptosis, and an uncontrolled cell cycle 149. The G-quadruplex (G4), a stable secondary structure formed by guanine-rich regions of DNA and RNA 150, inhibits the transcription of Myc by forming a stable structure within the Myc promoter 151. Gaikwad *et al.* suggested that the benzofuran scaffold D089 (MYC G-quadruplex ligand) induces cell cycle arrest at G1 phase and cell senescence and triggers pyroptosis in the multiple myeloma cell line L363 through cleavage mediated by caspase-1 instead of caspase-3 151, 152.

Preliminary evidence shows that docosahexaenoic acid (DHA), a long-chain polyunsaturated fatty acid, exerts inhibitory effects on cancer progression via the modulation of cell cycle arrest 153. For instance, DHA can suppress the proliferation of the pancreatic ductal adenocarcinoma cell line MIA PaCa-2 by cell cycle arrest at the G2/M phase and even trigger apoptosis 154. DHA is cytotoxic to the triple-negative breast cancer (TNBC) cell line MDA-MB-231 but has no harmful effects on the adjacent normal epithelial cell line MCF-10A. Additionally, this fatty acid leads to increased ASC levels and reduced pro-caspase-1 and pro-IL-1β levels in MDA-MB-231 cells, indicating pyroptosis initiation 155. Similarly, DHA also induces the cleavage of a key factor downstream of the caspase-1/GSDMD axis. However, the administration of YVAD (acetyl-tyrosyl-valyl-alanyl-aspartyl-chloromethylketone (ac-YVAD-CMK, a caspase-1 inhibitor) decreases pyroptosis-triggered membrane pore formation 155.

Intriguingly, the inflammasome serves as a double-edged sword in carcinogenesis. On the one hand, it initiates pyroptosis to eliminate cancer cells, and on the other hand, IL-1β generation facilitates cancer cell proliferation through autocrine signaling and limits the efficacy of 5-fluorouracil (5-FU) 156. Dumont and colleagues suggested that DHA reduces the 5-FU-induced production of IL-1β by inhibiting NLRP3 inflammasome assembly and the JNK signaling pathway in myeloid-derived suppressor cells (MDSCs), thereby improving the anticancer efficacy of 5-FU 157.

According to the available statistics, men are at higher risk of suffering from hepatocellular carcinoma (HCC) than women, and 17β-estradiol (E2) substantially contributes to this risk. Hence, E2 and its receptor play a protective role against the incidence of HCC 158. Wei suggested that E2 treatment could induce the upregulation of caspase-1 and IL-1β in the HCC cell line HepG2, which leads to NLRP3 inflammasome pyroptosis. NLRP3 can play an inhibitory role in cancer progression 159. However, it can also block autophagy through the downregulation of beclin-1, a decreased LC3 II/I ratio, and increased p62; additionally, the E2-triggered suppression of autophagy is necessarily dependent on the AMPK/mTOR signaling pathway 159.

In addition to small molecules, products derived from humans have the potential to induce pyroptosis. Emerging evidence has demonstrated that human umbilical cord mesenchymal stem cells (hUCMSCs) are a promising tool for cancer treatment. For instance, these cells can suppress the migration of the NSCLC cell line A549 and the HCC cell line BEL7402 160. Moreover, hUCMSCs possess some advantages compared with other types of MSCs, such as low immunogenicity and the harvesting of a large number of cells. It has been shown that hUCMSCs can trigger pyroptosis in the breast cancer cell line MCF-7 through the upregulation of NLRP1 and caspase-4, but these cells have no significant influence on the cell cycle 161.

Long noncoding RNAs (lncRNAs) refer to RNA sequences over 200 nucleotides in length without any corresponding encoded proteins 162. Emerging evidence has revealed that lncRNAs play a significant role in the modulation of biological functions, such as the cell cycle and apoptosis, through epigenetic regulation 163. Recent evidence suggests that lncRNAs can modulate tumorigenesis and pyroptosis. ΔNp63 is one of the isoforms of p63 and functions as an oncogene, in contrast to its counterpart TAp63, a tumor suppressor 164. ΔNp63 is upregulated by the lncRNA RP1‑85F18.6, which is overexpressed in CRC, and this overexpression indicates a poor prognosis. Ma demonstrated that the silencing of RP1‑85F18.6 by siRNA could result in increased GSDMD-N and pyroptosis in the colon adenocarcinoma cell line SW620, and this effect inhibits cancer cell proliferation, invasion and metastasis; hence, GSDMD is considered a favorable biomarker for patients with CRC 165.

Bromodomain-containing protein 4 (BRD4) belongs to the bromodomain and extraterminal domain (BET) protein family and plays a role in epigenetic regulation 166. Furthermore, it reportedly participates in several biological events, such as lysosomal function and autophagy 167. BRD4 is upregulated in the renal cell carcinoma (RCC) cell lines 786-O and ACHN but downregulates caspase-1. Jin and colleagues suggested that 786-O and ACHN cells treated with LPS and subsequently JQ-1 (bromodomain inhibitor) show relatively suppressed proliferation and metastasis due to the inhibition of BRD4 and the induction of NLRP3. Fortunately, JQ-1 exhibits no toxicity toward the normal human renal tubule epithelial cell line HK-2, and the inhibition of BRD4 by JQ-1 or genetic knockdown reportedly results in tumor growth suppression by pyroptosis in BALB/C nude mice injected with ACHN 168. Hence, the administration of JQ-1 or siRNA targeting BRD4 could be a promising strategy for RCC treatment through GSDMD-mediated pyroptosis.

Proline, glutamic acid, and leucine-rich protein 1 (PELP1) functions as an oncogenic protein and plays an important role in regulating the functions of hormone receptors (*e.g.*, estrogen receptor) 169. It has been shown that the overexpression of PELP1 and the activation of mTOR contribute to MCF-7 chemoresistance to tamoxifen. Hence, targeting PELP1 appears to be a promising strategy for cancer treatment 170. Furthermore, PELP1 expression is correlated with poor prognosis in patients with esophageal squamous cell carcinoma (ESCC), a cancer refractory to chemotherapy 171. Another study suggested that metformin could inhibit PELP1 to further induce GSDMD-related pyroptosis in the ESCC cell lines KYSE510 and KYSE140 and *in vivo* in immune-deficient mice inoculated with KYSE510 through the upregulation of miR-437 171.

Mammalian STE20-like kinase 1 (MST1), a core component of the Hippo signaling pathway, was originally considered a proapoptotic kinase that plays a crucial role in modulating cancer cell growth and proliferation 172. Recent evidence has demonstrated that MST1 is downregulated in colorectal cancer and is considered a potential biomarker for the early detection of CRC 173. Cui and colleagues indicated that MST1 induces pyroptosis through the upregulation of ROS in the pancreatic ductal adenocarcinoma (PDAC) cell lines BxPC-3 and FG. Overexpressed MST1 reportedly suppresses cancer cell proliferation, migration, and invasion. However, this increase could be attenuated by the administration of siMST1. Moreover, elevated levels of MST1 are positively correlated with the activation of caspase-1, caspase-3, and caspase-7 and the maturation of IL-1β and IL-18 174.

An experiment conducted by Teng indicated that polyphyllin VI (PPVI), which is derived from *Trillium tschonoskii Maxim* (TTM), a traditional Chinese medicine, activates the NLRP3 inflammasome in A549-bearing athymic nude mice. Moreover, PPVI promotes intracellular ROS generation and subsequently induces NF-κB activation. This effect contributes to increased cleavage of caspase-1 and GSDMD and ultimately leads to pyroptosis in the NSCLC cell lines A549 and H1299 175, 176.

LPS is reported to directly initiate noncanonical pyroptosis and further macrophage-mediated inflammation 177. Secretoglobin family 3A member 2 (SCGB3A2), also known as uteroglobin-related protein 1 (UGRP1), is a secreted protein that is predominantly expressed in epithelial cells of the airways and functions to induce pyroptosis by chaperoning LPS to the cytosol 178. Cell lines susceptible to cotreatment with SCGB3A2 and LPS express syndecan 1 (SDC-1), a cell surface receptor of SCGB3A20, and caspase-4 at high levels, which facilitates LPS-induced noncanonical pyroptosis; this finding also indicates the role of cotreatment in innate immunity. In contrast, the melanoma cell line B16F10 has little SDC-1 and is thus resistant to SCGB3A2 treatment 178, 179. Furthermore, SCGB3A2 reportedly induces tumor regression in C57BL/6 mice with murine Lewis lung carcinoma (LLC) cells 178. As shown in the study by Yokoyama *et al.*, SCGB3A2 and LPS administration induced pyroptosis and inhibited tumor growth by approximately 20% in the NSCLC cell lines NCI-H596, H358, H322, A549, and H157 and the CRC cell lines HCT116 and SW620. Thus, SCGB3A2 is considered a potential prognostic indicator for patients with lung adenocarcinoma 179.

The medication simvastatin is widely used to reduce cholesterol levels by inhibiting HMG-CoA reductase, reducing the risk of cardiovascular diseases 180. Simvastatin reportedly increases the sensitivity of the breast cancer cell line MCF-7 to doxorubicin through the upregulation of ROS and caspase-3. Moreover, this molecule reduces the level of Ras-related C3 botulinum toxin substrate 1 (Rac1), which is responsible for cell growth and motility and suppresses the expression of Cdk4/6 and cyclinD1 181. Recently, Wang *et al.* indicated that simvastatin inhibits the proliferation, migration, and invasion of the NSCLC cell lines H1299 and A549 by inducing pyroptosis, which was accompanied by elevated levels of NLRP3 and cleaved caspase-1 and the maturation of IL-1β and IL-18. Moreover, simvastatin can also suppress tumor growth via caspase-1-mediated pyroptosis in BALB/c-nude male mice injected with H1299 cells 182. Conversely, the administration of simvastatin with Ac-YVAD-CMK, an irreversible inhibitor of caspase-1, reduces the efficacy of simvastatin-induced pyroptosis, which rescues the viability of H1299 and A549 cells 182.

Val-boroPro, also known as talabostat, is a nonselective inhibitor of serine protease cleavage after proline residues. It reportedly induces immunostimulatory pyroptosis in macrophages and monocytes 183, 184. Emerging evidence shows that Val-boroPro can activate pro-caspase-1 and promote the cleavage of GSDMD instead of the apoptotic-related protein PARP through the inhibition of DDP8/DDP9 in the acute myeloid leukemia (AML) cell line MV4;11, which ultimately leads to caspase recruitment domain-containing protein 8 (CARD8)-induced pyroptosis. In addition, this effect has also been observed in female NSG mice injected with MV4;11-Luc Neo cells.

Nevertheless, the administration of talabostat does not result in the cleavage of pro-caspase-1 and the maturation of IL-1β, and the absence of these effects explains the slower induction of pyroptosis (days) compared with that of the canonical pyroptotic pathway (hours) (Table 4) 185.

Although GSDMD represents a double-edged sword in cancer progression, the activation of GSDMD is promising in cancer suppression. Here, we summarize several strategies, including small molecules, cells, and modified virus vectors, capable of inducing pyroptosis and with proven encouraging results in cancer treatment.

## Potential strategies targeting GSDME-mediated pyroptosis

Chemotherapies primarily exert antitumor effects by inducing caspase-3-mediated apoptosis. However, as mentioned above, several types of cancer exhibit rigorous chemoresistance by bypassing apoptosis. Caspase-3 is the common executor of apoptosis and GSDME-related pyroptosis. Therefore, the switch from apoptosis to pyroptosis appears to be a promising strategy for cancer treatment. The potential compounds targeting GSDME-related pyroptosis are described below (Table 5).

### Potential strategies targeting GSDME-mediated pyroptosis

The GSDME-mediated switch from apoptosis to pyroptosis has the potential to overcome antiapoptotic chemoresistance. Here, we summarize several strategies, including small molecules and nanomaterials, capable of inducing pyroptosis and with proven encouraging efficacy in cancer treatment.

Iron not only plays a crucial role in cellular homeostasis but also serves as an executor of ROS-mediated cell death, including apoptosis, necroptosis, and ferroptosis 186. Iron triggers apoptosis via the induction of mitochondrial outer membrane permeabilization (MOMP) and the release of cytochrome C, which facilitates the activation of caspase-3 and caspase-7 186. In addition, iron can initiate necroptosis and ferroptosis through the autophosphorylation of RIP1 and the peroxidation of phospholipids, respectively 187, 188. Nonetheless, iron-mediated pyroptosis has been less elucidated. Zhou and colleagues discovered that carbonyl cyanide m-chlorophenyl hydrazone (CCCP), a mitochondria-depolarizing agent, can promote iron-mediated ROS generation, which has the potential to trigger GSDME-dependent pyroptosis via the oligomerization of Tom20 (a mitochondrial outer membrane protein), the BAX-induced release of cytochrome C and the activation of caspase-3 in the melanoma cell line A375 and BALB/c nude mice injected with A375 cells 189.

Glioblastoma multiforme (GBM) is a lethal malignant tumor in the central nervous system due to chemo- and radioresistance. Unfortunately, the efficacy of pharmacotherapy is extremely limited by the blood-brain barrier (BBB). Galangin (GG, 3,5,7-trihydroxyflavone) is derived from *Alpinia officinarum*, a Chinese herbal medicine, and reportedly reverses chemoresistance to cisplatin in lung cancer by upregulating Bax and Bid and downregulating Bcl-2 190, 191. Kong and colleagues confirmed that GG promotes autophagic flux to suppress cancer cell proliferation, and increased MAP1 and LC3B-II levels and reduced SQSTM1 levels accompany this effect. Moreover, GGG also induces pyroptosis in the GBM cell lines U251 and U87MG and tumor-bearing male BALB/c athymic mice. GBM expresses GSDME at relatively high levels, and GG administration leads to elevated expression of the GSDME N-terminus and pyroptosis 192.

Lobaplatin (1,2-diamminomethylcyclobutane-platinum (II) lactate), a third-generation platinum anticancer drug, reportedly exhibits relatively lower toxicity and higher stability in water. Similar to other platinum drugs, lobaplatin can result in DNA cross-linking and eventually apoptosis. The combination of lobaplatin with docetaxel and epirubicin (both of which are widely prescribed to patients with breast cancer) significantly increases the complete pathological response and overall response rate in patients with TNBC 193. According to Yu *et al.*, lobaplatin triggers pyroptosis in the colon cancer cell lines HT-29 and HCT-116 by inducing the phosphorylation of JNK and ROS generation. Subsequently, this effect could lead to the activation of caspase-3 and the cleavage of GSDME instead of GSDMD, and this effect is accompanied by the overexpression of BAX 194. The colorectal adenocarcinoma cell line Caco-2 with deficient GSDME treated with lobaplatin shows a switch from pyroptosis to apoptosis. Similarly, lobaplatin-induced pyroptosis is also converted into apoptosis in GSDME-knockout BALB/c nude mice, but this conversion does not affect its inhibition of tumor formation 194.

MEK and BRAF inhibitors are targeted therapies for patients with *BRAF*^V600E/K^-mutant melanoma, but the tumor microenvironment determines their efficacy and efficiency, particularly T lymphocyte activation 195. The combination of MEK and the BRAF inhibitors mirdametinib and vemurafenib inhibits the MEK/ERK 1/2 signaling pathway, triggering Bim/Bmf-mediated mitochondrial depolarization. Subsequently, this effect leads to cytochrome C release, caspase-3 activation, and eventually GSDME-related pyroptosis in the mouse melanoma cell lines YUMM1.7 and D4M3.A and immunocompetent C57BL/6 mice. Moreover, the author proposed a model in which HMGB1 released from pyroptotic cells activates DCs and T cells as salvage therapies for patients with BRAFi + MEKi-resistant metastatic melanoma 114.

Metformin, a widely prescribed drug for patients with diabetes, reportedly activates AMPK and then its downstream signaling pathway 196. An experiment conducted by Zheng and colleagues indicated that metformin induces pyroptosis and elevated levels of LDH (an indication of pyroptotic cell cytotoxicity) in the HCC cell line HepG2 and the breast cancer cell line MCF-7 by activating AMPK and inducing mitochondrial dysfunction. Activated AMPK could trigger SIRT-1 and the production of NF-κB, which ultimately results in elevated expression of Bax and the release of cytochrome C. Thus, this process initiates the caspase-3-mediated cleavage of GSDME. Treatment with BAY11-7082 (an NF-κB inhibitor) suppresses pyroptosis induced by metformin in HepG2 and MCF-7 cells. Furthermore, metformin can lead to increased ROS generation within mitochondria and then positively facilitate the upregulation of Bax 197.

Miltirone, a lipophilic compound derived from *Salvia miltiorrhiza* Bunge, can inhibit platelet aggregation and be prescribed for patients with thrombotic diseases 198. Preliminary evidence shows that miltirone can induce apoptosis via the accumulation of intracellular ROS in the CRC cell line HCT116 199. Zhang *et al.* suggested that miltirone inhibits the phosphorylation of MEK and ERK1/2 and leads to the intracellular accumulation of ROS, which facilitates BAX-mediated caspase-3 activation and GSDME-induced pyroptosis in the mouse HCC cell line Hepa1-6 and in C57BL/6 male mice inoculated with Hepa1-6 cells 200.

Cotreatment with TNFα and cycloheximide (CHX) could switch the inflammatory response to apoptosis via the activation of caspase-8 201. In addition, preliminary evidence shows that TNFα and CHX can induce pyroptosis instead of apoptosis depending on the presence of GSDME 74, 202. GSDME is absent in several types of cancer (*e.g.*, colorectal cancer) due to promoter methylation 203. Nevertheless, GSDME can still be considered a potential target for these types of cancer with high GSDME expression. In the CRC cell line HCT116, which expresses high levels of GSDME but relatively low levels of GSDMD, the combination of TNFα and CHX could induce BAK/BAX, which leads to mitochondrial outer membrane permeabilization (MOMP) and GSDME-mediated pyroptosis through palmitoylation (covalent attachment of fatty acids) at the GSDME C-terminus (GSDME-C)^74^. However, this phenomenon could be inhibited by 2-bromopalmitate (2-BP) primarily through the suppression of palmitoylation on GSDME-C and increased interaction between GSDME-C and GSDME-N 74, 204.

Nanotechnology has been gradually applied in research and the clinic due to its advantages, such as lower toxicity to adjacent normal tissue, intracellular intake, and relatively prolonged presence in the body. Nanotechnologies consist of diverse types, such as liposomes and polymer-conjugated nanomedicine 205, 206. Zhao *et al.* designed a biomimetic nanoparticle (BNP) consisting of a polymeric core covered with the breast cancer membrane, ICG (indocyanine green), and DCT (decitabine), and showed that it induced the intracellular accumulation of calcium after photoactivation. This effect leads to mitochondrial damage and the activation of caspase-3, subsequently inducing GSDME-mediated pyroptosis in the tumors of 4T1 tumor-bearing mice 207. Interestingly, the administration of BNP also promotes the infiltration of CD11^+^ DCs, which triggers antigen presentation and immunotherapy 207.

Zhang *et al.* indicated that the abundance of GSDME expression is positively correlated with increased phagocytosis of tumor-associated macrophages and the infiltration of NK cells and CD8+ T cells 75. Erikes and colleagues revealed decreased infiltration of activated DCs and T cells in GSDME-deficient melanoma 114. As shown in a study by Zhao, nanoparticle-induced GSDME-mediated pyroptosis promotes the antigen presentation function of CD11+ DCs 207. In addition, cytokines released during pyroptotic cell death have been reported to induce innate and adaptive immunity. Little evidence corroborating the direct link of the strategies mentioned above to immunotherapy is available, but the induction of pyroptosis is a promising approach to aid in immunotherapy and deserves further investigation.

### Modulation of the switch between apoptosis and GSDME-mediated pyroptosis

The abundance of gasdermin proteins is the most significant determinant triggering the switch from apoptosis to pyroptosis 13, 15. GSDMC is specifically cleaved by caspase-8 to generate the N-terminus and form pores. Caspase-8 is also the upstream executor of caspase-3-mediated apoptosis. Hou *et al.* showed that death receptor-activated apoptosis switches to pyroptosis following the upregulation of GSDMC by the PD-L1/p-STAT3 axis 11. Similarly, noncanonical GSDME-mediated pyroptosis shares the same upstream signaling pathway with caspase-3-mediated apoptosis 70. Pyroptotic cell death occurs in cells with high GSDME expression. For instance, a study conducted by Yang revealed that cell lines expressing GSDME at high levels, including lung carcinoma PC-9, gastric carcinoma SGC-7901, and hepatoma Bel7402 cells, undergo pyroptotic cell death after treatment with cold atmospheric plasma (CAP), which promotes ROS generation 208.

Furthermore, nonsmall cell lung carcinoma H1299 cells with exogenous GSDME overexpression switch from apoptosis to pyroptosis after CAP administration 208. Another study conducted by Wang also indicated that overexpression of GSDME in HeLa cervical carcinoma cells contributes to the transformation from apoptosis to pyroptosis after treatment with 5-FU 76. The switch from apoptosis to pyroptosis potentially bypasses antiapoptotic obstacles and overcomes chemoresistance; thus, appropriate induction of GSDME-mediated pyroptosis is a primary issue in cancer treatment.

## Conclusions and perspectives

In this review, we first elucidate the cellular physiology of pyroptosis and introduce the inflammasome, and we then discuss the molecular mechanism of pyroptosis. Pyroptosis could be beneficial for the modulation of cancer progression primarily through two approaches. On the one hand, emerging evidence has shown that several cancer cell lines exhibit severe chemoresistance due to the impairment of apoptotic cell death; therefore, the induction of nonapoptotic cell death (*e.g.*, pyroptosis) appears promising for cancer treatment. On the other hand, pyroptosis is highly related to the immune response, and recent evidence indicates that the activation of pyroptosis could trigger a further immunostimulatory response within the tumor microenvironment. Furthermore, pyroptosis reportedly plays a significant role in promoting the efficacy of cancer immunotherapy. For instance, the upregulation of the gasdermin protein induces the infiltration of immune cells, such as M1 macrophages and CD4+ and CD8+ T lymphocytes, which increases the sensitivity of cells to anti-PD-1 mAbs.

Moreover, recent evidence has suggested that SARS-CoV-2 induces NLRP3/caspase-1-mediated pyroptosis in human monocytes, resulting in severe immune responses 209. Another experiment indicated that RNA viruses, such as encephalomyocarditis virus (EMCV), lead to potassium efflux from pyroptotic cells and subsequently activate the NLRP3 inflammasome in other cells 210. In contrast, Zika virus (ZIKV) contributes to neurodevelopmental disorders (*e.g.*, microcephaly) mainly by inducing pyroptosis in neural progenitor cells 211. Taken together, the appropriate management of viral infection might be a promising strategy for immunostimulation and conducive for cancer immunotherapy.

However, several urgent problems require further investigation. First, the induction of pyroptosis seems to be a potential strategy for the reactivation of immunity within the TME and suppression of cancer growth. Nevertheless, the development of pyroptosis inducers targeting cancer cells is the primary task because the immune response is systemic. In other words, improper control of pyroptosis would harm adjacent or other normal tissues instead of providing benefits to patients; hence, the disadvantages outweigh the advantages. Perhaps integrating specific antibodies or chemokine receptors on the surface of nanoparticles (NPs) containing pyroptosis inducers would enhance the trafficking of nanomaterials within the TME and more accurately target cancer cells. For instance, Liu *et al.* reported that several CXCR2 ligands (*e.g.*, CXCL2 and CCL2) are expressed at high levels in hepatocellular carcinoma cells, and the modification of CAR-T cells by introducing CXCR2 promotes trafficking and improves treatment 212. This strategy may be introduced into NPs containing pyroptosis inducers, but further investigations are needed.

Second, as mentioned above, the chemotherapy-induced switch from apoptosis to GSDME-mediated pyroptosis is a promising approach; nevertheless, GSDME expression is silenced in several types of cancer cells, such as prostate and breast cancer (https://www.proteinatlas.org/ENSG00000105928-GSDME/pathology). Fortunately, high GSDMD expression is observed in prostate and breast cancer (https://www.proteinatlas.org/ENSG00000104518-GSDMD/pathology); therefore, the activation of GSDMD-mediated pyroptosis is more conducive than the induction of GSDME-mediated pyroptosis. In addition, the GSDME content within cells is a key determinant of the switch between apoptosis and pyroptosis. Nevertheless, the expression of GSDME, its levels and which cellular conditions are required to trigger pyroptosis appear to be less well investigated. Therefore, an evaluation of gasdermin protein expression is needed in a clinical setting.

Third, the induction of pyroptosis exerts a remarkable effect on tumor regression, but preliminary studies have shown that pyroptosis induction is related to protumor effects and a poor prognosis. For instance, caspase-8/GSDMC-mediated pyroptosis results in a poor prognosis for patients with breast cancer 11. Additionally, Gao *et al.* indicated that the upregulation of GSDMD is positively correlated with TNM stages in patients with LUAD 140. Thus, further investigations in the clinical setting are needed to determine the specific role of pyroptosis in the treatment of different cancers.

Finally, noninvasive imaging has the potential to detect whether pyroptosis-related drugs accumulate within the TME. Magnetic particle imaging (MPI) is a noninvasive technique that directly detects the distribution of superparamagnetic iron oxide (SPIO) *in vivo*. MPI has been used to track adoptive T cells labeled with ferucarbotran (a clinically approved SPIO) *ex vivo* and evaluate the trafficking and persistence of adoptive T cells 213. Perhaps the same strategy can be performed using NPs containing pyroptosis inducers. This approach will enable physicians to receive information about the *in vivo* distribution and accumulation of nanomaterials using a noninvasive method.

## Figures and Tables

**Table 1:** Characteristics of apoptosis, necroptosis, and pyroptosis

Characteristics/Cell death	Apoptosis	Necroptosis	Pyroptosis
Pore formation on the cell membrane	No	Yes (executed by MLKL) 6	Yes (executed by gasdermin family member, N-terminal) 24
Formation of channels on the cell membrane	No	MLKL-formed pore is a selective ion channel 27	Gasdermin-formed pore is a nonselective channel 27
Plasma membrane blebbing	Yes (with apoptotic bodies)	No	Yes (with pyroptotic bodies) 28
Release of DAMPs from cells	No	Yes 16	Yes 12
Inflammatory response	No 18	Yes 16	Yes 19
Caspase	Caspase-dependent (caspase-2/-3/-6/-7/-8/-9/-10 are involved in the process) 214	Caspase-independent 6	Caspase-dependent (caspase-1/-3/-4/-5/-8/-11 are involved in the process) 9, 11
			Caspase-independent (granzyme A or granzyme B is involved in the process) 59, 75

**Table 2:** Summary of NOD-like receptors (NLRs) and the inflammasome

Inflammasome	Component			Activated by:	Ref.
	PRR (cytoplasmic)	ASC adaptor	Pro-caspase-1		
NLRP1 inflammasome	NLRP1	No	Yes	UVB and Bacillus anthracis toxins	40, 41, 46
NLRP3 inflammasome	NLRP3	Yes		PAMPs and DAMPs	37, 42
NLRC4 inflammasome	NLRC4	Yes/No		Flagella and TTSS expressed by Salmonella typhimurium	44, 47
AIM2 inflammasome	AIM2 Cytoplasmic dsDNA	Yes		Cytoplasmic dsDNA	37, 43
Pyrin inflammasome	Pyrin	Yes		Bacterial toxin-mediated inactivation of Rho GTPase	37, 45

**Table 3:** Introduction to gasdermin family members

Gene name	Executor protease	Cleavage site	Pore formation	Domains	Related disease	Ref.
*GSDMA*	NI	NI	Yes	N-terminal (pore-forming domain) and C-terminal (autoinhibitory domain)	Systemic sclerosis	62, 63
*GSDMB*	Granzyme A	Lys^244^/Lys^229^	Yes		Autoimmune disease (*e.g.*, asthma and Crohn's disease)	64, 65
*GSDMC*	Caspase-8	Asp240	Yes		MSI-H CRC and LUAD	11, 67, 68
*GSDMD*	Caspase-1	_272_FLTD_275_	Yes		PD	58, 69
	Caspase-4/-5/-11	_272_FLTD_275_				
*GSDME*	Caspase-3	Asp270	Yes		Nonsyndromic hearing impairment	70, 74, 75
	Granzyme B	Asp270				
*DFNB59*	NI	NI	No	N+ shorter C-terminal	Auditory neuropathy	56, 62, 77, 78

※ NI: Not identified.

**Table 4:** Potential strategies targeting GSDMD-related pyroptosis

Strategy	Mechanism	Cell line/*In vivo*	References
AAV1-P0-GSDMD N-terminus vector	Induction of GSDMD-mediated pyroptosis	Schwannoma cell lines NF2 and HEI-193	146
Benzofuran scaffold (D089)	Activation of caspase-1-mediated pyroptosis	Multiple myeloma cell line L363	151
DHA (docosahexaenoic acid)	Upregulation of ASC and activation of caspase-1	TNBC cell line MDA-MB-231	155
E2 (17β-estradiol)	Induction of NLRP3 inflammasome pyroptosis	HCC cell line HepG2	159
hUCMSCs	Upregulation of NLRP1 and caspase-4	Breast cancer cell line MCF-7	161
lncRP1‑85F18.6 siRNA	Promotion of GSDMD N-terminus	Colon adenocarcinoma cell line SW620	165
LPS and JQ-1	Inhibition of BRD4 and induction of NLRP3	RCC cell lines 786-O and ACHN	168
		BALB/C nude mice injected with ACHN	
Metformin	Inhibition of PELP1 and induction of GSDMD-mediated pyroptosis	ESCC cell lines KYSE510 and KYSE140	171
		Immune-deficient mice inoculated with KYSE510	
Polyphyllin VI (PPVI)	Promotion of intracellular ROS generation and induction of NLRP3 inflammasome pyroptosis	NSCLC cell lines A549 and H1299	176
SCGB3A2 and LPS	Induction of caspase-4-mediated pyroptosis	NSCLC cell lines NCI-H596, H358, H322, A549, and H157 and CRC cell lines HCT116 and SW620	178, 179
		C57BL/6 mice with murine LLC	
Simvastatin	Upregulation of NLRP3 and activation of caspase-1	NSCLC cell lines H1299 and A549	182
		BALB/c-nude male mice injected with H1299 cells	
Val-boroPro	Activation of caspase-1 and GSDMD to induce CARD8-mediated pyroptosis	Acute myeloid leukemia (AML) cell line MV4;11	185
		Female NSG mice injected with MV4;11-Luc Neo cells	

**Table 5:** Promising small molecules and nanomaterials targeting GSDME-related pyroptosis

Drug	Mechanism	Cell line/*In vivo*	Reference
**Small molecules**			
CCCP	Promotion of iron-mediated ROS generation, oligomerization of Tom20 and caspase-3-mediated pyroptosis	Melanoma cell line A375	189
		BALB/c nude mice injected with A375 cells	
Galangin (GG)	Upregulation of GSDME N-terminus	GBM cell lines U251 and U87 MG	192
		Tumor-bearing male BALB/c athymic mice	
Lobaplatin	Induction of the phosphorylation of JNK and ROS generation	Colon cancer cell lines HT-29 and HCT-116	194
		BALB/c nude mice	
Mirdametinib and Vemurafenib	Inhibition of the MEK/ERK 1/2 signaling pathway and promotion of Bim/Bmf-mediated mitochondrial depolarization	Mouse melanoma cell lines YUMM1.7 and D4M3.A	114
		Immunocompetent C57BL/6 mice	
Metformin	Activation of SIRT-1 and ROS-induced GSDME	HCC cell line HepG2 and breast cancer cell line MCF-7	197
Miltirone	Inhibition of the phosphorylation of MEK and ERK1/2 and promotion of the intracellular accumulation of ROS	Mouse HCC cells lines Hepa1-6	200
		C57BL/6 male mice inoculated with Hepa1-6	
TNFα and CHX	Upregulation of BAK/BAX, induction of MOMP and GSDME-mediated pyroptosis	CRC cell line HCT116	74, 204
**Nanomaterials**			
Designed BNP	Induction of mitochondrial damage and activation of caspase-3	4T1 tumor-bearing mice (*in vivo*)	207

## Abbreviations

E2: 17β-estradiol
α-NETA: 2-(α-naphthoyl)ethyl-trimethylammonium iodide
2-BP: 2-bromopalmitate
5-FU: 5-fluoruracil
AIM2: absent in melanoma 2
STAT3: signal transducer and activator of transcription 3
ALL: acute lymphocytic leukemia
AML: acute myeloid leukemia
AAV1: adeno-associated serotype 1 virus
AMPK: AMP-activated protein kinase
ADCC: antibody-dependent cell-mediated cytotoxicity
ASC: apoptosis-associated speck-like protein containing a CARD
BC: bladder cancer
BBB: blood-brain barrier
BMDC: bone marrow-derived DC
BET: bromodomain and extraterminal domain
BRD4: bromodomain-containing protein 4
CHOP: C/EBP homologous protein
CCCP: carbonyl cyanide m-chlorophenyl hydrazone
CARD8: caspase recruitment domain-containing protein 8
CAR-T: chimeric antigen receptor T-cell immune therapy
JNK: c-Jun N-terminal kinase
CAP: cold atmospheric plasma
CLRs: C-type lectin receptors
CHX: cycloheximide
CRS: cytokine release syndrome
CTLs: cytotoxic T lymphocytes
CTLA-4: cytotoxic T lymphocyte antigen 4
DAMPs: damage-associated molecular patterns
DCT: decitabine
DFNB59: deafness autosomal recessive 59
DCs: dendritic cells
DFNA5: deafness, autosomal dominant 5
DDP9: dipeptidyl peptidase 9
DHA: docosahexaenoic acid
EMCV: encephalomyocarditis virus
ESCRT: endosomal sorting complexes required for transport
EMT: epithelial-mesenchymal transition
ESCC: esophageal squamous cell carcinoma
ERK1/2: extracellular signal-regulated protein kinase 1/2
FKLC: familial keratosis lichenoides chronica
FMF: familial Mediterranean fever
FasL: Fas ligand
FADD: FAS-associated death domain protein
FAK: focal adhesion kinase
FIIND: function-to-find domain
GG: galangin
GSDMC: gasdermin C
GSDMD: gasdermin D
GSDME: gasdermin E
GBM: glioblastoma multiforme
GSH: glutathione
G4: G-quadruplex
GSDME-C: GSDME C-terminus
HCC: hepatocellular carcinoma
MSI-H CRC: high-frequency microsatellite instability colorectal cancer
HMGB1: high-mobility group protein 1
hUCMSCs: human umbilical cord mesenchymal stem cells
HIDS: hyperimmunoglobulinemia D syndrome
ICG: indocyanine green
IL-1R: IL-1 receptor
iBMDMs: immortalized bone marrow-derived macrophages
IFN-γ: interferon-γ
LDH: lactate dehydrogenase
LLC: Lewis lung carcinoma
LPS: lipopolysaccharide
lncRNAs: long noncoding RNAs
LUAD: lung adenocarcinoma
MPI: magnetic particle imaging
MHC: major histocompatibility complex
MST1: mammalian STE20-like kinase 1
MMPs: matrix metalloproteinases
MET: mesenchymal-epithelial transition
MOMP: mitochondrial outer membrane permeabilization
MLKL: mixed-lineage kinase domain-like pseudokinase
MSPC: multiple self-healing palmoplantar carcinoma
MDSCs: myeloid-derived suppressor cells
SIRT1: NAD-dependent deacetylase sirtuin-1
NPs: nanoparticles
NEK7: NIMA-related kinase 7
NLRP1 CT: NLRP1 C-terminal
NLRs: NOD-like receptors
NF-κB: nuclear factor-κB
PDAC: pancreatic ductal adenocarcinoma
PD: Parkinson's disease
PAMPs: pathogen-associated molecular patterns
PRRs: pattern recognition receptors
PA: phosphatidic acid
PI3K/Akt: phosphatidylinositol-3-kinase/protein kinase B
PS: phosphatidylserine
PARP: poly (ADP-ribose) polymerase
PPVI: polyphyllin VI
PCD: programmed cell death
PD-1: programmed cell death protein 1
PD-L1: programmed death ligand 1
PELP1: proline, glutamic acid, and leucine-rich protein 1
PKN1: protein kinase N1
PKN2: protein kinase N2
PYD: pyrin domain
Rac1: Ras-related C3 botulinum toxin substrate 1
ROS: reactive oxygen species
RCC: renal cell carcinoma
RLRs: retinoic acid-inducible gene 1 (RIG-1)-like receptors
SCGB3A2: secretoglobin family 3A member 2
SPIO: superparamagnetic iron oxide
SDC-1: syndecan 1
TLR: Toll-like receptor
TLR4: Toll-like receptor 4
TGF-β: transforming growth factor β
TGFBR2: transforming growth factor-beta receptor II
TTM: *Trillium tschonoskii* Maxim
TNBC: triple-negative breast cancer
TME: tumor microenvironment
TNF: tumor necrosis factor
TNFR: tumor necrosis factor receptor
UPR: unfolded protein response
UGRP1: uteroglobin-related protein 1
VbP: Val-boroPro
ZIKV: Zika virus
